# Cerebral activation following dynamic cycling in individuals with and without Parkinson's disease: an fNIRS investigation

**DOI:** 10.3389/fnhum.2026.1755116

**Published:** 2026-03-10

**Authors:** Brittany E. Smith, Lara M. Shigo, Julia Jones Huyck, Angela L. Ridgel

**Affiliations:** 1 Department of Exercise Science and Exercise Physiology, Kent State University, Kent, OH, United States; 2 Department of Sport and Health Sciences, Catawba College, Salisbury, NC, United States; 3 Brain Health Research Institute, Kent State University, Kent, OH, United States

**Keywords:** dynamic cycling, functional near-infrared spectroscopy, motor control, movement disorders, neurorehabilitation

## Abstract

**Introduction:**

High-cadence dynamic cycling has been associated with significant benefits on motor function in individuals with Parkinson's disease (PD). Despite clear improvements in motor symptoms in this population, the neurophysiological mechanisms are unknown. Functional near-infrared spectroscopy (fNIRS) is a neuroimaging tool that measures cortical activation by estimating hemoglobin content at the surface level of the brain.

**Methods:**

18 participants (*N* = 11 with PD) completed the present study in which changes in prefrontal cortical activity were investigated following high- and low-cadence dynamic cycling on the SMART bike, a motorized therapeutic stationary bicycle. fNIRS measures were acquired during finger tapping and cognitive assessment before and after dynamic cycling. Three-way mixed factorial ANOVA with repeated measures on time were conducted to determine differences in oxyhemoglobin concentrations within the prefrontal cortex (PFC) following dynamic cycling.

**Results:**

No significant differences were found in oxyhemoglobin responses. However, this is the first study in which researchers compared changes in fNIRS responses in people with PD (PwPD) and healthy age-matched controls following dynamic cycling on the SMART bike.

**Discussion:**

More work is warranted in larger sample sizes in order to continue the effort toward optimal exercise prescription for individuals with PD.

## 1 Introduction

The global prevalence of Parkinson's disease (PD) is expected to rise to 25.2 million individuals by Su et al. (2025). In the United States alone, approximately 90,000 individuals aged 65 and older are diagnosed each year (Willis et al., 2022). People with PD (PwPD) often experience classic motor symptoms of tremors, bradykinesia, postural instability, and rigidity (Dickson, 2018; Marras et al., 2018). Additionally, non-motor symptoms include hallucinations, sleep disorders, proprioceptive difficulties, in addition to deficits in working memory, executive function, and attention (Hausdorff et al., 2006; Possin et al., 2008; Elgh et al., 2009; Blandini, 2013; McKee and Hackney, 2013). Exercise-based therapies for motor and non-motor symptoms of PD have been extensively supported (Feng et al., 2020; Ellis et al., 2021; de Almeida et al., 2022; Gamborg et al., 2022; Ernst et al., 2023; Padilha et al., 2023; Yang et al., 2023; Langeskov-Christensen et al., 2024; Mitchell et al., 2024; Luthra et al., 2025).

High-cadence dynamic cycling is effective at improving motor symptoms of PwPD (Ridgel et al., 2012, 2013, 2015; Ridgel and Ault, 2019; Kim et al., 2024). High-cadence dynamic cycling is performed on the Speed Manipulated Adaptive Rehabilitation Therapy (SMARTbike) bicycle which uses a motor to assist PwPD with maintaining a high and variable cycling cadence, typically−80 revolutions per min (rpm) with second-by-second variabilities in motor speed + /− 4 rpm (Ridgel et al., 2009; Mohammadi-Abdar et al., 2016a,b).

In addition to improvements in motor function, treadmill training, resistance training, cycling, stretching, balance, and tango dancing have been shown to improve cognition in PwPD (McKee and Hackney, 2013; David et al., 2015; Picelli et al., 2016). PwPD have reduced overall cerebral blood flow compared to age-matched control participants (Lavy et al., 1979; Globus et al., 1985), which may be associated with cognitive deficits, cerebral atrophy, and dopamine deficiencies ADDIN EN.CITE (Pelizzari et al., 2020; Li et al., 2023)Exercise in PwPD has been linked to improvement in motor and cognitive function via increases in activation of the prefrontal cortex (PFC) region (Shin et al., 2024).

Functional near-infrared spectroscopy (fNIRS) is a non-invasive neuroimaging tool which measures changes in oxyhemoglobin concentration (ΔHbO_2_) and cortical activation during motor and cognitive tasks, allowing for examination of mechanisms underpinning symptom improvement (Maidan et al., 2016; Thumm et al., 2018; Stuart et al., 2019; Pelicioni et al., 2020; Hofmann et al., 2021; Bonilauri et al., 2022; Schejter-Margalit et al., 2022). fNIRS has been used to examine changes in cerebral oxygenation following acute and chronic exercise in both healthy and clinical populations (Moriarty et al., 2019, 2020; Liao et al., 2021). It is important to note that fNIRS does not directly measure cerebral blood flow. However this information can be inferred due to the oxygenated hemoglobin concentration of the tissue (Pinti et al., 2020). Collectively, fNIRS studies propose that exercise training at moderate intensity may be associated with improved neural efficiency, as shown by a reduced oxygen requirement during cognitive tasks in cardiovascular disease, older adult, and healthy populations (Moriarty et al., 2019, 2020; Liao et al., 2021).

Moriarty et al. (2020) showed significant increases in ΔHbO_2_ within the left prefrontal cortex (LPFC) during cognitive testing in a sample of patients following 6 weeks of cardiac rehabilitation and these increases were negatively associated with cognitive function (Moriarty et al., 2020). While the findings of Moriarty et al. (2020) cannot be extrapolated to PwPD, it is important to acknowledge the previous fNIRS work in clinical populations due to the limited literature. Liao et al. (2021) showed significant reductions in bilateral PFC ΔHbO_2_ concentration during cognitive tasks following 12-weeks of an exergaming and exercise intervention consisting of resistance, aerobic, and balance training. Moriarty et al. (2019) also compared responses in PFC activity during cognitive testing following various conditions including high-intensity exercise, moderate-intensity exercise, yoga exercise, and a non-exercise control in a sample of healthy adults (Moriarty et al., 2019). Moderate-intensity exercise resulted in significantly higher hemoglobin difference in LPFC compared to the other intensities, with no differences in cognitive performance, although LPFC activation did show a significant negative relationship with processing speed (Moriarty et al., 2019).

fNIRS has also been utilized to evaluate PFC activation in PwPD (Maidan et al., 2018; Hoang et al., 2022). Hoang et al. evaluated PFC activity changes during walking following 5 weeks of exercise rehabilitation training in PwPD. There was a significant reduction in right PFC (RPFC) activation following the exercise program (Hoang et al., 2022). Maidan et al. (2018) acquired fNIRS measurements of the PFC during usual and complex walking tasks following 6 weeks of treadmill training and treadmill training plus virtual reality in a sample of 64 PwPD. Both interventions studied were associated with reduced PFC activation (Maidan et al., 2018). Additionally, the reduced number of falls following the intervention were associated with reduced PFC activation (Maidan et al., 2018). Maidan et al. (2018) suggests that exercise training may lead to increases in neural plasticity and more efficient neural recruitment. Reduced PFC activation during walking suggests improvement of automaticity of gait in PwPD following the intervention, with improvements augmented with the addition of sensory feedback to exercise (Maidan et al., 2018; Hoang et al., 2022).

While previous SMARTbike dynamic cycling studies have documented significant improvements in motor function, the potential neurophysiological mechanisms of this exercise modality are unknown in PwPD. The purpose of this study was to investigate changes in PFC ΔHbO_2_ concentrations in PwPD and age-matched (± 3 years) healthy controls without PD following high- (80 rpm) and low-cadence (60 rpm) dynamic cycling. ΔHbO_2_ concentrations were measured during a finger tapping motor task and a cognitive Trail Making Test (TMT). All participants (PwPD and healthy controls) performed both conditions of dynamic cycling to compare differences in the dose-response effect of speed variability, as well as to provide further context into neurophysiological changes in PwPD compared to normal aging. We hypothesized that high-cadence dynamic cycling would be associated with greater changes in ΔHbO_2_ concentrations within the PFC compared to low-cadence dynamic cycling in PwPD and healthy age-matched control participants.

## 2 Materials and methods

**Figure 1:**
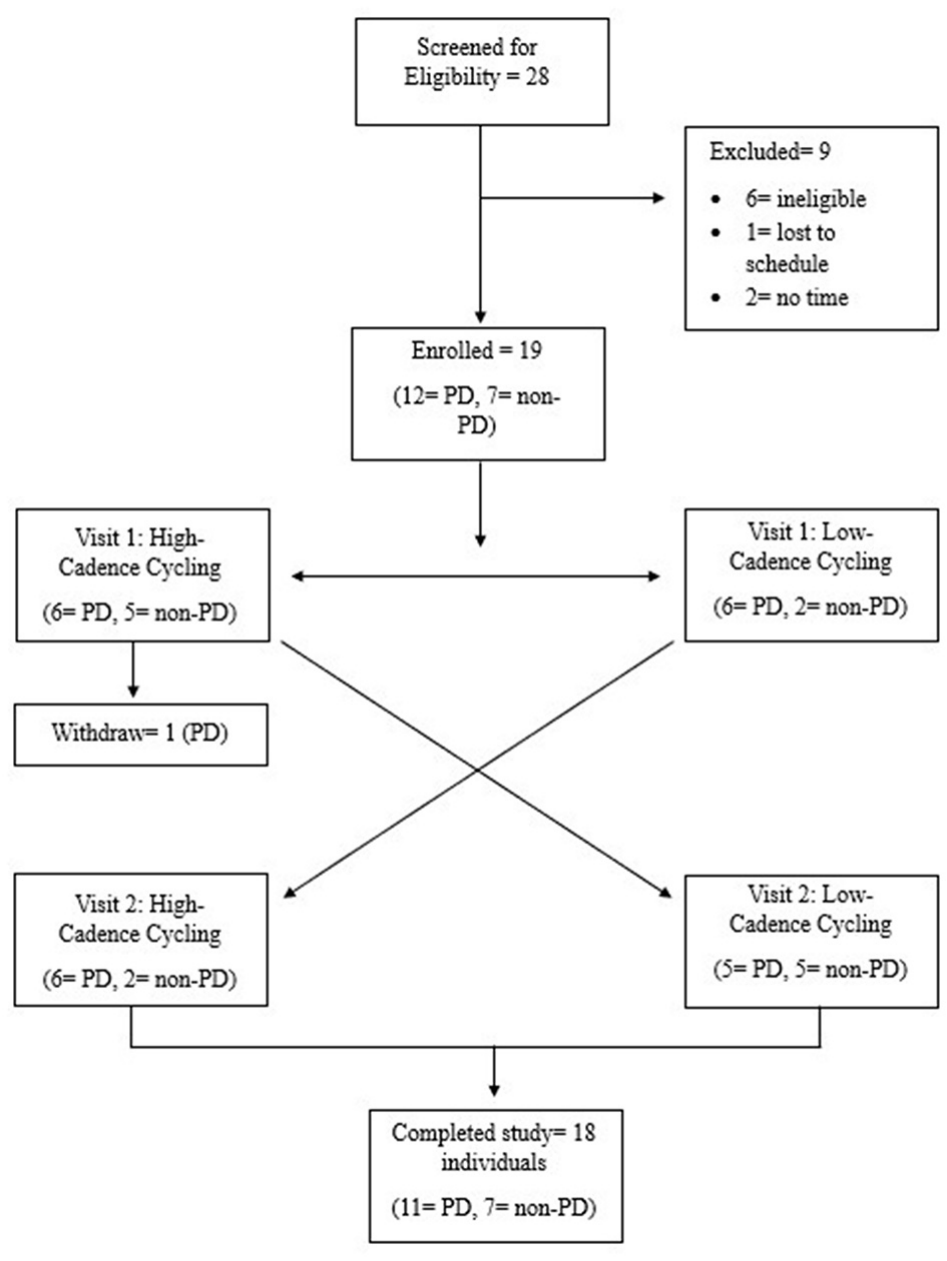
Consort diagram.

Participants reported to the Kent State University Brain Health Research Institute in Kent, Ohio for two visits, separated by 7 days. Visits were scheduled at the same time of day to minimize variations in motor function and cortical activation. All participants were asked to keep nutrition intake, physical activity, and medication timing consistent across both visits. PwPD were ON levodopa medication at both study visits. At visit one, all participants gave written informed consent and were pre-screened with the American College of Sports Medicine Preparticipation Questionnaire to rule out underlying disease aside from PD and document any signs or symptoms of cardiovascular disease (American College of Sports Medicine, 2022). PwPD must have received a formal PD diagnosis from a physician to be eligible. PwPD participants were randomized to either low-cadence dynamic cycling at 60 revolutions per min (rpm) or high-cadence dynamic cycling (80 rpm) for visit one. Randomization was conducted using a random number generator (1-100) publicly available on the internet. If the random number generator yielded an odd number, the participant would complete low-cadence dynamic cycling at visit one. If the random number generator yielded an even number, the participant would complete high-cadence dynamic cycling at visit one. At visit one, 8 total participants (six PwPD and two non-PD) performed low-cadence dynamic cycling. Non-PD participants were age-matched (±3 years) to a PwPD and performed the opposite cycling cadence order for counterbalancing to minimize carryover effects. Figure 1 depicts a consort diagram of study recruitment and protocol. Prior to each dynamic cycling session, all participants completed fNIRS cortical activation (ΔHbO_2_) measurements using the multichannel Brite (Artinis Medical Systems B.V.) system. Baseline motor function was measured with the Kinesia ONE (Great Lakes Neurotechnologies (Cleveland, Ohio). The Kinesia ONE is an objective assessment tool for motor symptom severity that has been validated in PD and essential tremor (ET) populations (Giuffrida et al., 2009; Heldman et al., 2011; Mera et al., 2012). The Kinesia ONE sensor is worn on the index finger and heel during various motor tasks that assess resting tremor, postural tremor, kinetic tremor, dyskinesia, gait/freezing of gait, leg agility, and bradykinesia. The motor data is then analyzed with algorithms developed by Great Lakes Neurotechnologies to score symptom severity (0-4) similar to the Movement Disorder Society Unified Parkinson's Disease Rating Scale Part III (MDS-UPDRS-III) (Giuffrida et al., 2009; Heldman et al., 2011; Mera et al., 2012).

Participants were fitted with an appropriately sized snug neoprene cap based on their head circumference. Study staff followed Artinis' recommended procedures to ensure signal quality within the OxySoft software. Data collection utilized a sampling rate of 25 hertz (Hz) and transmitting wavelengths were−760 and−850 nanometers (nm). The Polhemus Patriot (Polhemus Incorporated, Colchester, Vermont) was utilized for optode digitization. Because the larger study investigated changes in both the prefrontal cortex (PFC) and sensorimotor cortex (SMC) regions, authors utilized the 1 x 9 + 1 x 13 channel template (22 channels) and the standard inter-optode distance of 30 mm for optode placement. The 1 x 13 sub-template was utilized for the PFC measurements reported in this manuscript. ΔHbO2 concentrations in channels Rx1 – T x 1, R x 1 – T x 3, R x 2 – T x 1, R x 2 – T x 3, R x 2 – T x 4, and R x 4 – T x 3 were averaged together for the right prefrontal cortex (RPFC). Channels R x 4 – T x 8, R x 6 – T x 4, R x 6 – T x 8, R x 6 – T x 9, R x 8 – T x 8, and R x 8 – T x 9 were averaged together for the left prefrontal cortex (LPFC). BrainVoyager Brain Tutor (Rainer Goebel) software and the 10/20 anatomical correlation system were used to measure the proper regions of interest. All fNIRS data was processed using a low-pass (0.5 Hz) filter to remove noise due to changes in HR, BP, or movement. Data was recorded, managed, and analyzed in OxySoft Version 4.0 (Artinis Medical Systems B.V.).

fNIRS measurements were performed during a finger tapping motor task and during Trail Making Test Part A (TMT A) and B (TMT B) cognitive assessments to evaluate both motor and cognitive cerebrovascular responses. The finger tapping task protocol evaluated changes in cerebral blow flow and connectivity using fNIRS during single finger tapping, following protocol of Khan et al. (2021). The task consisted of 2-min of rest followed by 10 s of thumb tapping on a flat table surface with hand at rest, followed by 10 s of rest, followed by subsequent finger tapping of each finger with 10 s of rest in between. The finger tapping protocol was repeated for a total of three trials. All participants performed this task with the dominant hand and were instructed to perform the tapping at a medium-to-fast pace with their eyes closed throughout the duration of the measurement. fNIRS collected ΔHbO_2_ throughout the rest and active portions of the task.

TMT A and TMT B, assessments of executive function, began with an initial 2-min rest, followed by completion of each task and an additional 30-s rest period. Participants were instructed to keep their eyes closed during the rest portions of the task, allowing for a baseline PFC activity measure and were able to open their eyes once study staff cued to begin the TMT. fNIRS measured ΔHbO_2_ during rest and active portions of the task. ΔHbO_2_ was calculated as the relative concentration during each task minus 30 s of baseline HbO_2_ (Moriarty et al., 2020). Hemodynamic responses have been found to stabilize within 15-20 s in populations with neurodegenerative disease (Wang et al., 2024). 30 s of baseline was utilized in the present study to assure return to resting values as well as accounting for filtering of changes due to respiration and heart rate, per Artinis recommendations. The 30-s prior to baseline served as the normalization period and was omitted from the analysis. Additionally, the time to completion and errors were recorded for each TMT, indicating cognitive performance. Faster times and fewer errors represented better cognitive performance.

Dynamic cycling consisted of a 5-min warm up (60 rpm), 30-min of exercise at assigned cadence (60 or 80 rpm), and a 5-min cool-down (60 rpm). The dynamic motor of the SMARTbike assists the rider in maintaining the required cadence throughout the exercise session. Although the SMARTbike is motorized, dynamic cycling is not passive. The rider is required to exert effort onto the pedals during the exercise session and is given visual feedback on the touch screen display to determine if effort is sufficient. Heart rate (HR) and rating of perceived exertion (RPE) were measured throughout all dynamic cycling sessions. Follow-up fNIRS measures were performed 30-min post dynamic cycling to control for increases in ΔHbO_2_ due to anticipated normal physiological changes in HR and blood pressure following exercise and subsequent recovery. Speed and power data were collected second-by-second on the SMART bike and exported for analysis with a sample entropy (SamEn) MATLAB script developed by Mohammadi-Abdar (Richman and Moorman, 2000; Mohammadi-Abdar et al., 2016a,b; Gates, 2021). SamEn was utilized for this analysis due to previous associations with improvement in motor function and to continue investigations into its utility for exercise prescription for PwPD (Kim et al., 2024). Previous studies have indicated that motor symptom improvement is linked to greater SamEn of cadence (Ridgel et al., 2013, 2015; Kim et al., 2024). Entropy of cadence was analyzed to evaluate associations with ΔHbO_2_ responses in the PFC across high- and low-cadence conditions.

Statistical analyses were performed with SPSS Version 28 (IBM SPSS, Chicago, IL) with an alpha level of 0.05. Descriptive statistics were used to compare groups across demographics. Three-way mixed factorial ANOVA (two conditions by two time points by two groups) with repeated measures on condition and time were conducted to determine significant differences in ΔHbO_2_ responses. If significant three-way interactions were found, separate two-way ANOVAs were run for each group (PwPD and non-PD). If two-way ANOVA results were significant, paired samples *t*-tests were run to compare pre- and post- values across conditions. Pearson correlation analyses were performed to assess relationships between ΔHbO_2_ concentrations and cognitive performance measures. Given the small sample size, *post hoc* sensitivity analyses were conducted using G^*^Power Version 3.1 to determine estimates of power. For the two-way ANOVAs, utilizing α = 0.05 and 80% power, we found that the sensitivity to effects was *f* = 0.70 [($\eta_{\text{p}}^{2}$ = 0.33)]. Additionally, given the sample size, α = 0.05, and 80% power, the three-way ANOVAs yielded a sensitivity effect of *f* = 0.448 ($\eta_{\text{p}}^{2}=$ 0.17).

## 3 Results

### 3.1 Descriptives

A total of 18 participants (11 PwPD, 7 non-PD, 61-78 years) completed the study (Table 1). All non-PD participants were matched to a PwPD. Prior to recruitment, the required sample size for this study calculated by GPower (Version 3.1) yielded 14 participants (7 PwPD; 7 non-PD). The sample size calculation utilized data from a previous study (Kim et al., 2024) which utilized MDS-UPDRS-III change score data for PwPD who performed dynamic cycling (*d* = 1.75). The effect size was calculated based on unpublished data from Kim et al. (2024) using the UPDRS-III change score means of−5.3 ± 3.70 (Patient-Specific Adaptive Dynamic Cycling group; *n* = 13) and + 4.2 ± 6.72 (Active Control group; *n* = 10). Additional participants were recruited for a more robust sample size and to account for withdrawals. One PwPD withdrew following visit one due to health complications. There were no significant differences in age, height, weight, or body mass index (BMI) between groups (Table 1). Baseline Kinesia ONE motor scores were averaged for PwPD (17.9 ± 5.4). When compared to the MDS-UPDRS-III cut-off values, the PwPD group in this study is classified as mild PD (< 32) (Skorvanek et al., 2017). Baseline TMT A completion times for all participants were compared to normative values according to age (Tombaugh, 2004). Baseline TMT A percentile rankings were conservatively classified based on >12 years of education and were not significantly different between groups (PwPD: 56.0 ± 24.0%, Non-PD: 60 ± 26.7%, *p* = 0.745).

**Table 1:** Participant demographic information.

	**PD (*N* = 11)**	**Non-PD (*N* = 7)**	***p*-value**
Age (years)	68.6 ± 5.4	70.6 ± 3.5	0.397
Height (m)	1.7 ± 0.1	1.8 ± 0.1	0.099
Weight (kg)	69.0 ± 15.2	82.1 ± 13.0	0.078
BMI (kg/m^2^)	23.8 ± 3.8	25.6 ± 2.9	0.285
Baseline TMT A Percentile (%)	56.0 ± 24.0	60 ± 26.7	0.745
Kinesia ONE Motor Score	17.9 ± 5.4	N/A	N/A
LEDD (mg)	459.5 ± 328.3	N/A	N/A
PD duration (years)	3.09 ± 2.63	N/A	N/A

Values are reported as mean ± standard deviation. BMI, Body Mass Index; LEDD, levodopa equivalent daily dose; m, meters; kg, kilograms; mg, milligrams.

### 3.2 Cycling variables

Table 2 displays the average dynamic cycling variables for all subjects. All variables except HR (*p* = 0.489) were significantly different across dynamic cycling conditions (high- vs. low-cadence). High-cadence dynamic cycling resulted in increased RPE, power, and SamEn of cadence and power compared to low-cadence dynamic cycling. RPE, effort (%), and power were significantly different between the two dynamic cycling cadences in the PwPD, but not in non-PD participants (Table 3).

**Table 2:** Average dynamic cycling variables.

	**High-Cadence (80 rpm)**	**Low-Cadence (60 rpm)**	***p*-value**
Cadence (rpm)	78.7 ± 1.1	59.6 ± 0.4	**< 0.001**
HR (bpm)	84.3 ± 12.2	82.7 ± 10.4	0.489
RPE	12.5 ± 1.7	10.7 ±2.0	**< 0.001**
Effort (%)	82.2 ± 33.4	96.5 ± 11.9	**0.042**
Power (W)	24.6 ± 12.4	13.3 ± 15.6	**< 0.001**
SamEn of Cadence	1.1 ± 0.3	0.9 ± 0.1	**0.019**
SamEn of Power	1.8 ± 0.13	1.7 ± 0.2	**0.006**

Values are reported as mean ±standard deviation. HR, Heart Rate; bpm, beats per minute; RPE, Rating of Perceived Exertion; W, Watts; SamEn, Sample Entropy. Bold values indicate *p* < 0.05.

**Table 3:** Average dynamic cycling variables for PD group.

	**High-Cadence (80 rpm)**	**Low-Cadence (60 rpm)**	***p*-value**
Cadence (rpm)	78.4 ± 1.4	59.4 ± 0.2	**< 0.001**
HR (bpm)	83.0 ± 12.7	82.2 ± 11.1	0.792
RPE	**12.7** **±1.4**	**11.0** **±2.2**	**< 0.001**
Effort (%)	**71.3** **±39.4**	**94.3** **±15.1**	**0.043**
Power (W)	**6.5** **±14.4**	**20.7** **±10.2**	**< 0.001**
SamEn of Cadence	1.0 ± 0.4	0.8 ± 0.1	0.122
SamEn of Power	1.8 ± 0.1	1.6 ± 0.3	0.061

Values are reported as mean ±standard deviation. HR, Heart Rate; RPE, Rating of Perceived Exertion; SamEn, Sample Entropy. Bold values indicate *p* < 0.05.

**Figure 2:**
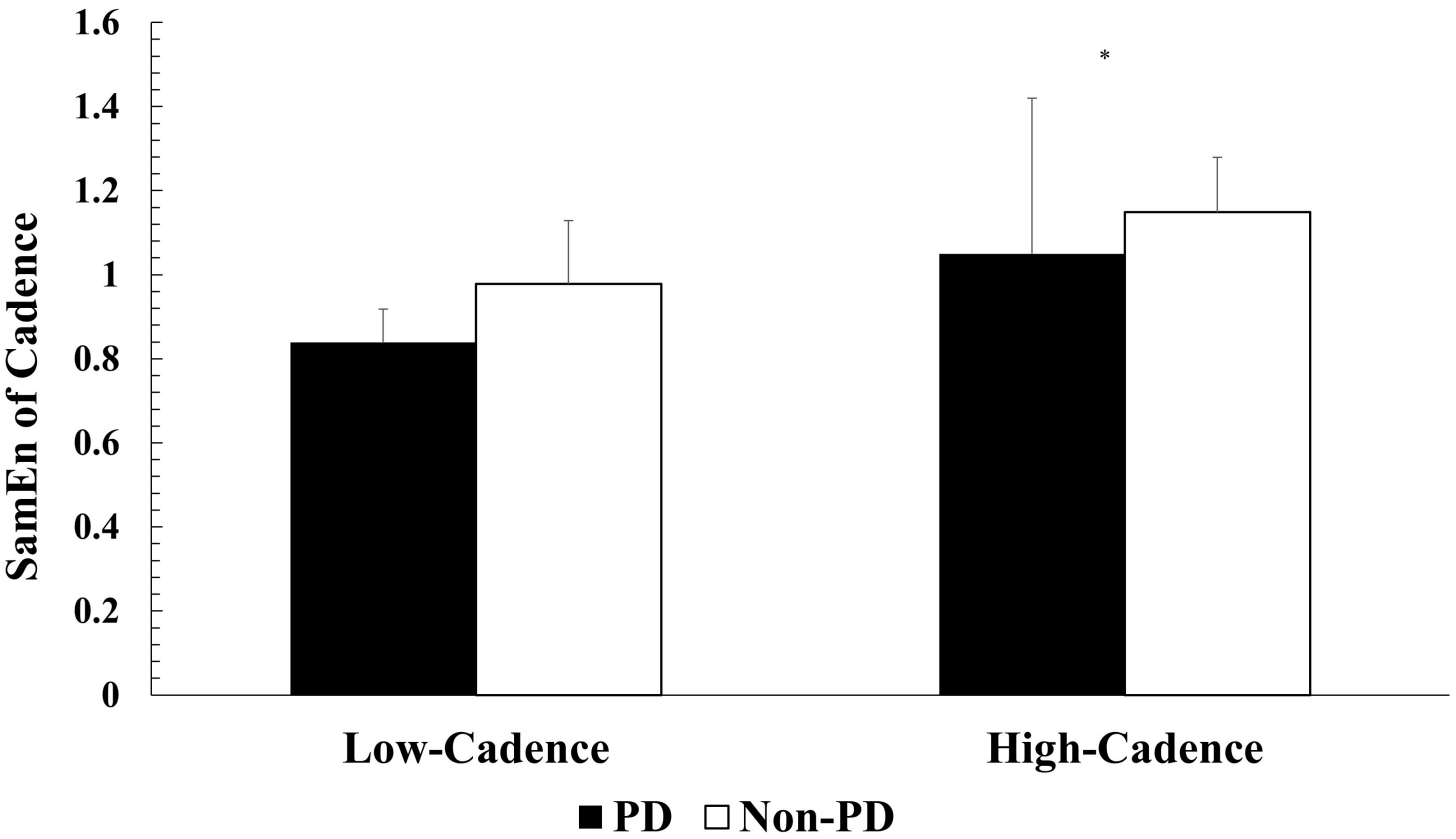
Mean SamEn of cadence across dynamic cycling conditions. Values are reported as mean ± standard deviation. * *p* < 0.05.

Two-way ANOVA (cadence x group) was utilized to compare SamEn of cadence across dynamic cycling cadences and participant groups. There was no significant interaction in SamEn between cadence and group (*F* = 0.067, *p* = 0.800, $\eta_{\text{p}}^{2}$ = 0.004, 1-β = 0.057). However, there was a significant main effect of cadence (*F* = 6.037, *p* = 0.027, $\eta_{\text{p}}^{2}$ = 0.287, 1-β = 0.632), indicating that SamEn was significantly greater in both groups during high-cadence dynamic cycling (PwPD, 1.05 ± 0.37; non-PD, 1.15 ± 0.13) compared to low-cadence dynamic cycling (PwPD, 0.84 ± 0.08; non-PD, 0.98 ± 0.15) (Figure 2).

### 3.3 Primary outcomes

#### 3.3.1 ΔHbO_**2**_ results during TMT

Statistical analyses found no significant differences in ΔHbO_2_ concentration within the RPFC or LPFC areas during TMT A and B following either condition of dynamic cycling. Three-way mixed factorial ANOVA found no significant interaction (cadence x group x time) on ΔHbO_2_ concentration within the RPFC (*F* = 0.104, *p* = 0.751, $\eta_{\text{p}}^{2}$ = 0.006, 1-β = 0.061) and left PFC (LPFC) (*F* = 0.199, *p* = 0.661, $\eta_{\text{p}}^{2}$ = 0.012, 1-β = 0.070) during TMT A. Additionally, no significant interaction was found during TMT B within the RPFC (*F* = 0.045, *p* = 0.834, $\eta_{\text{p}}^{2}$ = 0.003, 1-β = 0.055) or LPFC (*F* = 0.268, *p* = 0.612, $\eta_{\text{p}}^{2}$ = 0.016, 1-β = 0.078). A main effect of cadence was trending toward significance for ΔHbO_2_ during TMT B within the LPFC (*F* = 3.238, *p* = 0.091, $\eta_{\text{p}}^{2}$ = 0.168, 1-β = 0.394). No other significant main effects or two-way interactions were found. When comparing non-significant mean percent (%) ΔHbO_2_ concentrations, RPFC in PwPD experienced a 39% (Pre: 1.29 ± 1.175; Post: 0.781 ± 0.808) (Range:−106% to + 205%) and 11% (Pre: 1.155 ± 0.756; Post: 1.033 ± 1.306) (Range:−103% to + 101%) decrease during TMT A following low-cadence and high-cadence dynamic cycling, respectively. The non-PD group showed a mean reduction of 19% (Pre: 1.253 ± 1.049; Post: 1.007 ± 1.012) (Range:−94% to + 1721%) and 7% (Pre: 1.362 ± 1.378; Post: 1.269 ± 0.884) (Range:−-512% to + 45%) following low-cadence and high-cadence dynamic cycling respectively within the RPFC during TMT A. In LPFC, PwPD group showed a 23% (Pre: 1.610 ±1.121; Post: 1.228 ±0.974) (Range:−58% to + 67%) drop in ΔHbO_2_ concentration following low-cadence and a < 1% (Pre: 1.405 ± 0.809; Post: 1.393 ± 1.305) (Range:−92% to + 389%) reduction following high-cadence. Non-PD showed a 4% (Low-cadence range:−553% to + 91%; High-cadence range:−7410% to 399%) (Low-cadence Pre: 1.242 ± 1.005; Post: 1.177 ± 0.961) (High-cadence Pre: 1.446 ± 1.766; Post: 1.390 ± 1.113) decrease following both cycling conditions during TMT A. ΔHbO_2_ concentration showed reductions within the RPFC in both groups following both exercise conditions during the TMT B (PwPD: 7% following low-cadence, Pre: 1.800 ± 1.607; Post: 1.657 ± 1.693; Range:−75% to + 494%; 3% following high-cadence, Pre: 1.663 ± 1.377; Post: 1.612 ± 1.191; Range:−67% to + 399%; non-PD: 14% following low-cadence, Pre: 1.771 ± 1.275; Post: 1.513 ± 1.512; Range:−89% to + 128%; 19% following high-cadence, Pre: 1.710 ± 1.711; Post: 1.369 ± 1.124; Range:−259% to + 208%). Within the LPFC during TMT B, the PwPD showed a mean increase of 11% (Pre: 2.207 ± 1.831; Post: 2.465 ± 2.096) (Range:−91% to 589%) following low-cadence, while displaying a 10% (Pre: 2.059 ±1.436; Post: 1.854 ± 1.278) (Range:−103% to 130%) reduction following high-cadence. Non-PD showed a 13% and 19% decrease in ΔHbO_2_ following low-cadence (Pre: 1.952 ± 1.400; Post: 1.688 ± 1.592) (Range:−83% to + 191%) and high-cadence during TMT B (Pre: 1.498 ± 1.652; Post: 1.209 ± 1.282) (Range:−179% to + 95%).

### 3.4 Correlations

#### 3.4.1 ΔHbO_2_ and cognitive performance

**Figure 3:**
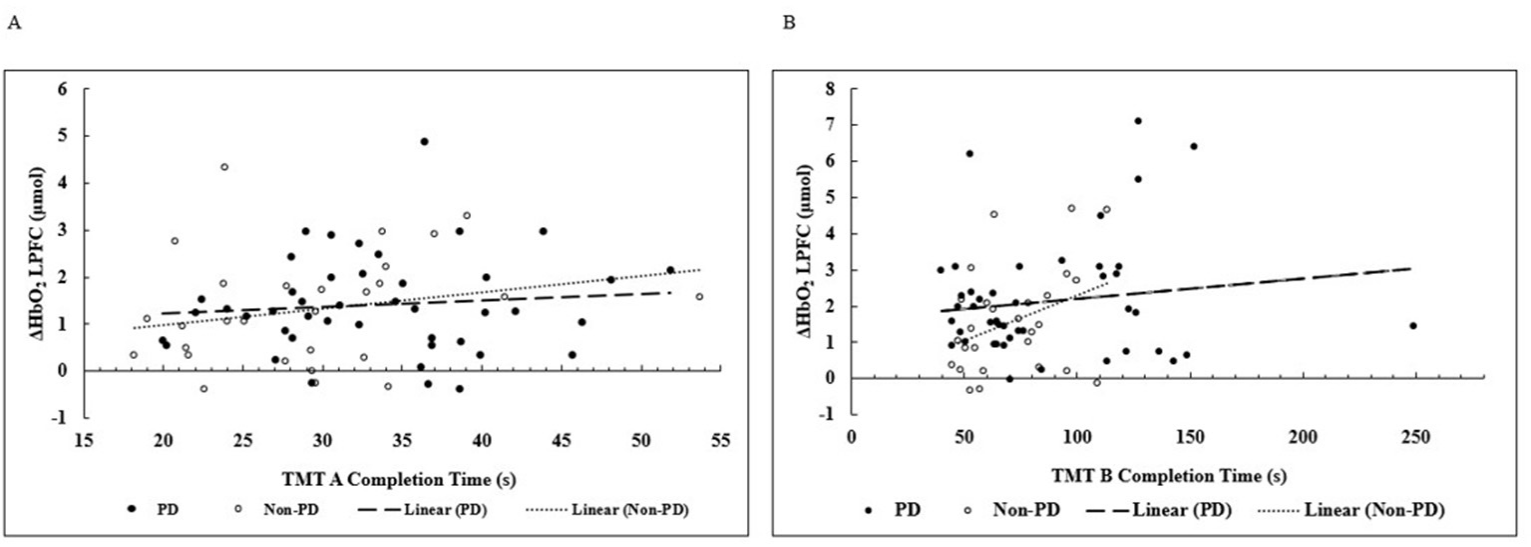
**(A)** Relationship between ΔHbO_2_ concentration within the LPFC during TMT A and TMT A Completion Time **(B)** Relationship between ΔHbO_2_ concentration within the LPFC during TMT B and TMT B Completion Time.

Correlation analysis revealed no statistically significant relationships between LPFC ΔHbO_2_ concentration and cognitive performance, as measured by TMT A and TMT B completion times. Figure 3 displays the correlation analysis between LPFC ΔHbO_2_ concentration and TMT A and TMT B completion times. Pearson correlation showed a non-significant, positive correlation between LPFC ΔHbO_2_ concentration and TMT A completion time when all participants were analyzed together (*r* = 0.160, p = 0.180) (Figure 3A). When analyzed separately, the PwPD group showed a non-significant, positive correlation between TMT A completion time to LPFC ΔHbO_2_ concentration (*r* = 0.098, *p* = 0.527). The non-PD group showed a similar trend (*r* = 0.229, *p* = 0.241).

When correlating ΔHbO_2_ concentration within the LPFC to TMT B completion time performance, there was a non-significant, positive correlation with grouped data (*r* = 0.214, *p* = 0.071), in PwPD only (*r* = 0.140, *p* = 0.366), and non-PD only (*r* = 0.362, *p* = 0.058) (Figure 3B). It is important to acknowledge the large variability in responses. Given the nature of the disease, the wide variation in responses to exercise is not uncommon in PwPD (Ridgel et al., 2015; Kim et al., 2024). For example, one PwPD participant's TMT performance (−250 s) was almost 100 s longer than the next closest PwPD participant.

#### 3.4.2 ΔHbO_2_ results during finger tapping

**Figure 4:**
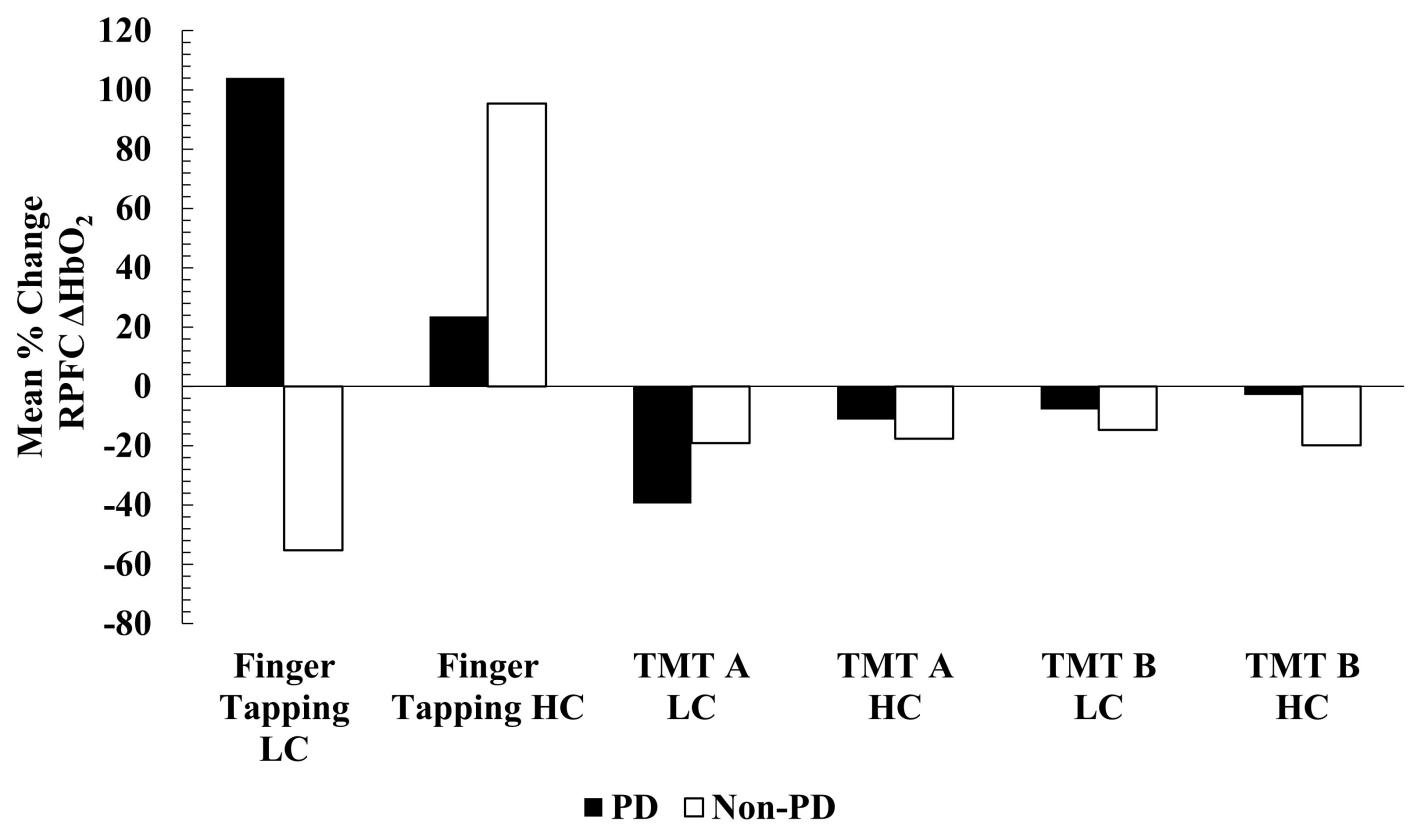
Mean % change RPFC ΔHbO2 concentration. LC, low-cadence dynamic cycling; HC, high-cadence dynamic cycling.

No statistically significant effects in ΔHbO_2_ concentration were found within RPFC or LPFC during the finger tapping motor task across both groups and conditions, however mean data suggests potentially greater reliance on HbO_2_ within the PFC during the motor task. Three-way mixed factorial ANOVA found no significant interaction on ΔHbO_2_ concentration within the RPFC (df = 1.550, *F* = 1.267, *p* = 0.291, $\eta_{\text{p}}^{2}$ = 0.073, 1-β = 0.225) or LPFC (df = 1.427, *F* = 1.362, *p* = 0.268, $\eta_{\text{p}}^{2}$ = 0.078, 1-β = 0.230) during finger tapping across groups or time. For these three-way ANOVAs, Greenhouse- Geisser corrections were reported due to violation of Mauchly's Test of Sphericity for time and the cadence x time interaction (*p* < 0.001). No significant main effects or two-way interactions were found. All trials of finger tapping were averaged for all participants; however, for one participant, only one trial was used due to the incorrect hand being used for the other trials. When looking at mean responses, ΔHbO_2_ concentrations within the RPFC non-significantly increased during all individual (thumb, index, middle, ring, pinky, etc.) finger tapping trials following low-cadence dynamic cycling while non-PD participants showed decreases. However, following high-cadence dynamic cycling, mean ΔHbO_2_ increased within the RPFC during individual finger tapping trials in all participants. When all ΔHbO_2_ concentrations were averaged across all fingers, PwPD showed a mean increase of 103% (Pre: 0.391 ± 0.086; Post: 0.798 ± 0.123) following low-cadence dynamic cycling within the RPFC (Range: + 62% to + 141%), while the non-PD group showed a mean 55% reduction (Pre: 0.493 ±0.168; Post: 0.220 ±0.106) within the RPFC following low-cadence dynamic cycling (Range:−81% to−13%). After high-cadence dynamic cycling, PwPD showed a 23% increase (Pre: 0.301 ± 0.066; Post: 0.373 ± 0.099) in ΔHbO_2_ during finger tapping within the RPFC (Range: + 11% to + 43%), while non-PD displayed an averaged 95% increase (Pre:−0.181 ± 0.183; Post:−0.008 ± 0.090) (Range: + 46% to + 167%). Figure 4 displays the mean % changes in RPFC ΔHbO_2_ concentration.

**Figure 5:**
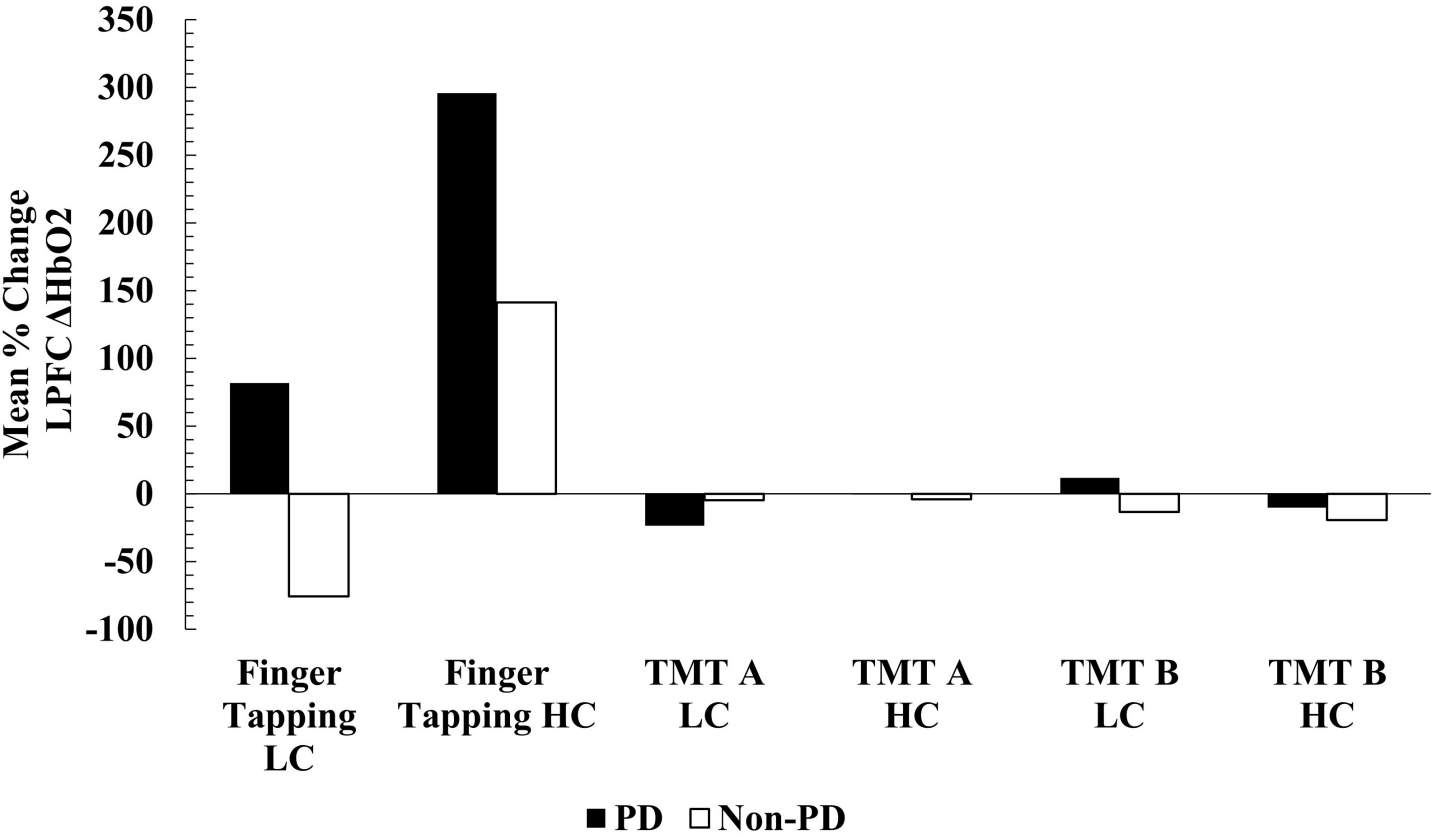
Mean % change LPFC ΔHbO2 concentration. LC, low-cadence dynamic cycling; HC, high-cadence dynamic cycling.

Mean ΔHbO_2_ concentration within the LPFC during individual finger tapping trials non-significantly increased in PwPD following low cadence cycling, however, non-PD showed decreased in LPFC responses. Following high-cadence dynamic cycling, LPFC responses were similar to RPFC for both participant groups. When finger tapping trials were averaged for LPFC following high-cadence dynamic cycling, PwPD participants showed increases in mean ΔHbO_2_ concentration by almost 300% (Pre: 0.056 ± 0.066; Post: 0.222 ± 0.089) (Range: + 117% to + 1076%) and non-PD showed close to a 142% increase (Pre:−0.241 ± 0.151; Post: 0.099 ± 0.058) (Range: + 114% to + 264%). Following low-cadence dynamic cycling, PwPD showed mean LPFC increases by 81% (Pre: 0.242 ± 0.078; Post: 0.439 ± 0.121) (Range: + 45% to + 155%), while non-PD showed a decrease of almost 76% (Pre: 0.448 ± 0.104; Post: 0.109 ± 0.089) (Range:−94% to−38%). Minor reductions in ΔHbO_2_ concentration were found in the LPFC during TMT for both groups except for PwPD during the TMT B following low cadence cycling. Figure 5 displays the mean % changes in LPFC ΔHbO_2_ concentration. Although ΔHbO_2_ responses were not statistically significant in the present study across groups or time, the mean results and trends suggest potential greater oxygen requirement in the PFC during motor tasks and greater efficiency, or lesser oxygen requirement, during cognitive tasks in PwPD.

### 3.5 Secondary outcomes

#### 3.5.1 TMT completion time

Lastly, secondary outcome statistical analysis revealed significant differences in TMT A completion time between groups. Non-PD completed TMT A significantly faster following low-cadence dynamic cycling compared to PwPD. Three-way mixed factorial ANOVA found no significant interaction (group x cadence x time) on TMT A completion performance time (*F* = 0.700, *p* = 0.415, $\eta_{\text{p}}^{2}$ = 0.042, 1-β = 0.123). There was a significant main effect of time (*F* = 5.052, *p* = 0.039, $\eta_{\text{p}}^{2}$ = 0.240, 1-β = 0.560) and a significant two-way group x time interaction (*F* = 4.677, *p* = 0.046, $\eta_{\text{p}}^{2}$ = 0.226, 1-β = 0.529). Independent samples *t*-tests showed significantly different TMT A completion times following low-cadence cycling between groups (*t* = 2.564, *p* = 0.021). PwPD completed the test in 34.4 ± 7.2 s and non-PD completed in 26.0 ± 5.9 s. Faster completion times indicate more efficient cognitive performance. While TMT A performance did not show any significant change in PwPD, there was a mean overall reduction in PFC ΔHbO_2_ concentration following dynamic cycling. This suggests that following exercise, PwPD were able to maintain cognitive performance with a reduced oxygen requirement within the PFC.

Three-way mixed factorial ANOVA found no significant interaction (group x cadence x time) for TMT B performance time (*F* = 0.414, *p* = 0.529, $\eta_{\text{p}}^{2}$ = 0.025, 1-β = 0.093). No significant main effects or two-way interactions were found. Non-PD group showed mean improvements in TMT B completion time following each cycling cadence; however, PwPD only saw a mean improvement following low-cadence cycling.

## 4 Discussion

Despite a lack of statistically significant findings for primary outcomes of ΔHbO_2_ responses, this study is novel in that responses in PFC activation as measured with fNIRS have not previously been evaluated in a dynamic cycling intervention in neither PwPD nor non-PD individuals. These results add to the limited literature however, the large degree of variability in responses in ΔHbO_2_ and TMT performance in PwPD likely contributed to the lack of significance. Nevertheless, this variability is consistent with previous exercise investigations in this population (Ridgel et al., 2015; Kim et al., 2024). Although not statistically significant, there was an overall mean reduction in LPFC and RPFC ΔHbO_2_ concentrations in both groups following exercise, except during TMT B following low-cadence dynamic cycling in PwPD. During finger tapping, the PFC showed an overall increased mean response when all fingers were combined for PwPD, while non-PD showed a reduction in RPFC following both cadence and in the LPFC following low-cadence dynamic cycling. While a larger sample size is needed to confirm responses, these initial data suggest that there is a potential increase in PFC activity during a motor task and a decrease in PFC activity during cognitive tasks following dynamic cycling in PwPD compared to the non-PD group which saw increases and decreases in PFC activity during a motor task following each high-cadence and low-cadence dynamic cycling, respectively. Like PwPD, PFC activity was also reduced during the cognitive task in the non-PD participants. Given that findings are opposite of what was originally expected, we propose that the direction of results in the present study may suggest increased neural efficiency as a potential mechanism following dynamic cycling exercise. In the present study, despite a lack of statistical significance, task performance was similar across time points however, mean cerebral activation was lower.

Other studies have explored the changes in cerebral blood flow within the PFC using fNIRS before, during, and/or after exercise in populations of healthy individuals and PwPD (Maidan et al., 2016; Thumm et al., 2018; Moriarty et al., 2019; Liao et al., 2021; Ding et al., 2022). However, contrary findings and differences in study protocols warrant the need for additional investigations, particularly in PwPD. The current study aimed to provide a clearer picture into fNIRS methodology and activation responses to exercise within the PFC in PwPD. Maidan et al. compared frontal lobe activation in various walking tasks (usual, dual-task, and obstacle negotiation) in PwPD and healthy older adults. Results showed that PwPD symptom severity was negatively associated with PFC HbO2 concentration during usual walking (*r* = −0.280, *p* = 0.022) and obstacle negotiation walking (*r* = −0.355, *p* = 0.003) (Maidan et al., 2016). During usual walking, PwPD showed greater increases in HbO2 compared to healthy older adults, as well as significantly worse performance. While the current study was not a training intervention, results suggest the potential goal to improve neural efficiency in PD by reducing the activation load on the PFC to enhance motor function through exercise (Maidan et al., 2016). Thumm et al. investigated PFC activation using fNIRS during overground and treadmill walking in PwPD. 80% of the participants showed reductions in HbO2 during treadmill walking compared to overground walking (Thumm et al., 2018). These results differ in a meaningful way, as the present study utilized a dynamic cycling protocol specifically designed for PwPD. The SMART bike relies on a motor to maintain cadence while still requiring effort to be exerted onto the pedals, ensuring a synergistic relationship between the SMART bike and rider with continually varying stimuli. Although similar modalities have not been evaluated, studies have examined the impact of exercise intensity on PFC activity (Moriarty et al., 2019). Moriarty et al. conducted a small study (*n* = 8) in adults (age 18-44 years) in which PFC oxygenation was compared during cognitive tasks following various exercise intensities. The greatest cerebral oxygenation during cognitive testing followed moderate intensity aerobic exercise (Moriarty et al., 2019). Additionally, across all exercise intensities, there was a significant negative correlation between LPFC oxygenation and cognitive processing speed (Moriarty et al., 2019). These results show differing results compared to the present study in terms of neural efficiency and potential lower requirement for cerebral oxygen following high-cadence dynamic cycling although Moriarty et al. (2019) investigated changes in oxygenation within a sample of physically active individuals with a mean age of 35 years and who were free from any cardiovascular, metabolic, pulmonary, behavioral, psychological, or neural disorder. Liao et al. performed a training intervention using fNIRS to measure cortical activation in frail older adults, who were not diagnosed with PD. Liao et al. utilized two training interventions: exergaming and combined exercise, which consisted of aerobic, resistance, and balance exercises. Both groups completed 12-weeks of their randomized intervention (3 times per week for 60 min) (Liao et al., 2021). fNIRS measurement was performed during the Montreal Cognitive Assessment (MoCA) assessment across the bilateral PFC. Both groups improved their MoCA performance, although exergaming, the condition with consistent feedback showed greater improvement (Liao et al., 2021). Both groups also displayed reductions in RPFC and bilateral PFC activation following the 12-week intervention, while exergaming showed reduction in LPFC as well (Liao et al., 2021). These results agree with the present study in PwPD, indicating increased neural efficiency (reductions in HbO_2_ concentration with improvements in cognitive function) in this population of older adults following an exercise paradigm including sensory biofeedback. Similarly, a training intervention by Ding et al. compared changes in brain activity using fNIRS following 6-weeks of treadmill exercise training in 19 PwPD and 19 healthy controls during normal and dual-task walking. PwPD displayed slower gait speed, shorter stride, and lower cognitive scores compared to control participants (Ding et al., 2022). Additionally, PwPD had lower resting cortical activation at baseline however, following 6-weeks of training, PwPD displayed similar resting activation to control participants (Ding et al., 2022). Following training, PFC activation in PwPD was significantly reduced during the walking tasks, but lower in normal walking (Ding et al., 2022). Results from Ding et al. (2022) may suggest potential neural mechanisms following treadmill training in which PFC activation is reduced and further widens the brain's capacity to efficiently perform complex tasks, improving motor function and symptoms in PwPD (Ding et al., 2022), which aligns with results of the present study.

PwPD showed a slight reduction in cognitive flexibility and set shifting compared to non-PD individuals, which is similar to what others have reported (Cools et al., 2001; Monchi et al., 2004). Cools et al. reported that when compared to control participants, PwPD exhibit deficits in guided task-set switching and were more susceptible to interference during a cognitive assessment which required the ability to switch between letter and digit-naming (Cools et al., 2001). Monchi et al. utilized functional magnetic resonance imaging (fMRI) to assess cortical activity in PwPD and age-matched control participants during a computerized Wisconsin Card Sorting Task (WCST). PwPD showed greater cortical activation in the prefrontal regions during the task however, PwPD completed a lower number of trials of the WCST, as well as committed more errors compared to the control participants (Monchi et al., 2004). When comparing changes in cognitive flexibility following exercise in PwPD, Duchesne et al. found no improvements in cognitive flexibility (TMT) after a 3-month high-intensity cycling program, despite improvements in cognitive inhibition (Stroop Test) (Duchesne et al., 2015). Based on these findings, it is likely that variability in exercise training may have differential effects on cognitive flexibility. It is reasonable to acknowledge that TMT B does possess increased complexity compared to TMT A and may not improve following a single exercise session in PwPD.

Prior to the present study, no dynamic cycling interventions had compared SamEn across low or high cycling cadences. This is the first study to compare SamEn of cadence values between a single session of 60 rpm and 80 rpm exercise in PwPD and non-PD individuals. These findings are novel, as no previous study utilizing the SMARTbike or similar equipment has compared SamEn of these two cadences. However, given the compelling evidence of the SamEn on motor symptom improvement, this comparison indicates the value of high-cadence dynamic cycling. fNIRS results vary compared to some previous literature however, SMARTbike exercise contrasts to the traditional, non-dynamic exercise modalities that have primarily been studied.

SamEn of cadence has been shown to be associated with motor symptom improvement in PwPD (Kim et al., 2024). This study demonstrates that SamEn of cadence is greater during a single session of high-cadence dynamic cycling at 80 rpm, which further advocates for faster paced exercise in PwPD. The lack of significant differences in heart rate when comparing the two exercise cadences suggests that cardiovascular stress was similar between the two conditions, even though there were differences in effort and power.

## 5 Limitations

There are several limitations to the present study. The small sample size and large variability in responses limits our ability to draw specific conclusions of the investigation. A larger number of study participants are needed to achieve appropriate statistical power for a significant three-way interaction (group x cadence x time). The null findings of the current study should be interpreted with caution, as the small and underpowered sample size may have limited ability to detect statistically significant effects. Additionally, this study only examined acute effects following dynamic cycling. Longer interventions may be required to see significant responses in the outcome measures in PwPD, as one single session may not have provided sufficient stimulus to elicit significant responses in ΔHbO_2_ of PFC and cognitive performance. Unlike our single session study, previous fNIRS exercise studies in PD have utilized longer exercise intervention (5-6 weeks) and more complex motor tasks such as walking (Maidan et al., 2018; Ding et al., 2022; Hoang et al., 2022). It is possible that differences in muscle recruitment or movement complexity produce different responses in PFC activation. The current study also utilized a randomization protocol to control trial order; however, condition balancing was not implemented. Lastly, we acknowledge limitations with the fNIRS protocol and processing of the present study. fNIRS responses were averaged across task completion times for each participant. Future researchers are recommended to model data to address variations in completion times and avoid discrepancies. Additionally, the current study protocol consisted of a 30-s baseline period in which HbO_2_ concentrations were averaged. This method may introduce multiple slow oscillations which reduce the signal-to-noise ratio and limit the ability to detect task-related changes. Future studies should consider employing shorter baseline resting periods (5-10 s) to improve hemodynamic data response quality.

Despite the lack of significant major findings, a strength of the present study was the pre- and post- dynamic cycling fNIRS measures at all research visits. Performing measurements pre- and post- at all sessions helped to account for potential confounding variables that may influence cortical activation across visits such as medication, sleep quality and arousal state, alterations in HR and blood pressure, and nutrition. Future studies should also investigate differences in responses across PwPD of differing severities, Hoehn and Yahr (H & Y) stages, or levodopa equivalent daily dosages (LEDD). Future researchers may consider monitoring physiological variables post-exercise to further ensure similar physiological states as pre-exercise fNIRS measurements, as well as short-separation channels to parcel out superficial blood flow changes from true tissue oxygenation responses from exercise.

Overall, no significant changes in PFC activation were observed during motor or cognitive tasks following an acute bout of low- and high-cadence dynamic cycling in a small sample of PwPD and healthy, age-matched control participants. Despite lack of statistically significant findings, this study provides valuable preliminary and transparent insight into fNIRS methodology, which is currently limited following exercise in PwPD. More work is needed to investigate the role of dynamic cycling on ΔHbO_2_ concentration within the PFC, as well as cognitive performance. Wide variation in responses to exercise within the PwPD population demonstrates the need for future studies to continue development toward appropriate exercise prescriptions for optimal motor symptom improvement.

## Data Availability

The raw data supporting the conclusions of this article will be made available by the authors, without undue reservation.

## References

[B1] American College of Sports Medicine, (2022). American College of Sports Medicine's Guidelines for Exercise Testing and Prescription. Philadelphia, PA: Wolters Kluwer.

[B2] Blandini F. (2013). Neural and immune mechanisms in the pathogenesis of Parkinson's disease. J. Neuroimmune Pharmacol. 8, 189–201. doi: 10.1007/s11481-013-9435-y 23378275

[B3] Bonilauri A. Sangiuliano Intra F. Rossetto F. Borgnis F. Baselli G. Baglio F. (2022). Whole-head functional near-infrared spectroscopy as an ecological monitoring tool for assessing cortical activity in Parkinson's disease patients at different stages. Int. J. Mol. Sci. 23:14897. doi: 10.3390/ijms232314897 36499223 PMC9736501

[B4] Cools R. Barker R. A. Sahakian B. J. Robbins T. W. (2001). Mechanisms of cognitive set flexibility in Parkinson's disease. Brain 124, 2503–2512. doi: 10.1093/brain/124.12.2503 11701603

[B5] David F. J. Robichaud J. A. Leurgans S. E. Poon C. Kohrt W. M. Goldman J. G. . (2015). Exercise improves cognition in Parkinson's disease: the PRET-PD randomized, clinical trial. Mov Disord. 30, 1657–1663. doi: 10.1002/mds.26291 26148003 PMC4609235

[B6] de Almeida F. O. Santana V. Corcos D. M. Ugrinowitsch C. Silva-Batista C. (2022). Effects of endurance training on motor signs of parkinson's disease: a systematic review and meta-analysis. Sports Med. 52, 1789–1815. doi: 10.1007/s40279-022-01650-x 35113386

[B7] Dickson D. W. (2018). Neuropathology of Parkinson disease. Parkinsonism Relat. Disord 46, S30–s33. doi: 10.1016/j.parkreldis.2017.07.033 28780180 PMC5718208

[B8] Ding H. Droby A. Anwar A. R. Bange M. Hausdorff J. M. Nasseroleslami B. . (2022). Treadmill training in Parkinson's disease is underpinned by the interregional connectivity in cortical-subcortical network. NPJ Parkinson's Dis. 8:153. doi: 10.1038/s41531-022-00427-3 36369264 PMC9652466

[B9] Duchesne C. Lungu O. Nadeau A. Robillard M. E. Boré A. Bobeuf F. . (2015). Enhancing both motor and cognitive functioning in Parkinson's disease: aerobic exercise as a rehabilitative intervention. Brain Cogn. 99, 68–77. doi: 10.1016/j.bandc.2015.07.005 26263381

[B10] Elgh E. Domellöf M. Linder J. Edström M. Stenlund H. Forsgren L. (2009). Cognitive function in early Parkinson's disease: a population-based study. Eur. J. Neurol. 16, 1278–1284. doi: 10.1111/j.1468-1331.2009.02707.x 19538208

[B11] Ellis T. D. Colón-Semenza C. DeAngelis T. R. Thomas C. A. Hilaire M. S. Earhart G. M. . (2021). Evidence for early and regular physical therapy and exercise in parkinson's disease. Semin. Neurol. 41, 189–205. doi: 10.1055/s-0041-1725133 33742432 PMC8678920

[B12] Ernst M. Folkerts A. K. Gollan R. Lieker E. Caro-Valenzuela J. Adams A. . (2023). Physical exercise for people with Parkinson's disease: a systematic review and network meta-analysis. Cochrane Database Syst. Rev. 1:Cd013856. doi: 10.1002/14651858.CD013856.pub2 36602886 PMC9815433

[B13] Feng Y. S. Yang S. D. Tan Z. X. Wang M. M. Xing Y. Dong F. . (2020). The benefits and mechanisms of exercise training for Parkinson's disease. Life Sci. 245:117345. doi: 10.1016/j.lfs.2020.117345 31981631

[B14] Gamborg M. Hvid L. G. Dalgas U. Langeskov-Christensen M. (2022). Parkinson's disease and intensive exercise therapy - an updated systematic review and meta-analysis. Acta Neurol. Scand. 145, 504–528. doi: 10.1111/ane.13579 34997759

[B15] Gates P. (2021). Development of a model to predict outcomes after dynamic cycling people with Parkinson's disease (Doctoral dissertation). Columbus, OH: Kent State University.

[B16] Giuffrida J. P. Riley D. E. Maddux B. N. Heldman D. A. (2009). Clinically deployable Kinesia technology for automated tremor assessment. Mov. Disord. 24, 723–730. doi: 10.1002/mds.22445 19133661

[B17] Globus M. Mildworf B. Melamed E. (1985). Cerebral blood flow and cognitive impairment in Parkinson's disease. Neurology 35, 1135–1139. doi: 10.1212/WNL.35.8.1135 4022347

[B18] Hausdorff J. M. Doniger G. M. Springer S. Yogev G. Simon E. S. Giladi N. (2006). A common cognitive profile in elderly fallers and in patients with Parkinson's disease: the prominence of impaired executive function and attention. Exp. Aging Res. 32, 411–429. doi: 10.1080/03610730600875817 16982571 PMC1868891

[B19] Heldman D. A. Giuffrida J. P. Chen R. Payne M. Mazzella F. Duker A. P. . (2011). The modified bradykinesia rating scale for Parkinson's disease: reliability and comparison with kinematic measures. Mov. Disord. 26, 1859–1863. doi: 10.1002/mds.23740 21538531 PMC3324112

[B20] Hoang I. Ranchet M. Cheminon M. Derollepot R. Devos H. Perrey S. . (2022). An intensive exercise-based training program reduces prefrontal activity during usual walking in patients with Parkinson's disease. Clin. Parkinsonism Relat. Disord. 6:100128. doi: 10.1016/j.prdoa.2021.100128 34988428 PMC8704467

[B21] Hofmann A. Rosenbaum D. Int-Veen I. Ehlis A. C. Brockmann K. Dehnen K. . (2021). Abnormally reduced frontal cortex activity during trail-making-test in prodromal Parkinson's disease-a fNIRS study. Neurobiol. Aging 105, 148–158. doi: 10.1016/j.neurobiolaging.2021.04.014 34087607

[B22] Khan H. Noori F. M. Yazidi A. Uddin M. Z. Khan M. N. A. Mirtaheri P. (2021). Classification of individual finger movements from right hand using fNIRS signals. Sensors 21;23. doi: 10.3390/s21237943 34883949 PMC8659988

[B23] Kim Y. Smith B. E. Shigo L. M. Shaikh A. G. Loparo K. A. Ridgel A. L. (2024). Utilizing entropy of cadence to optimize cycling rehabilitation in individuals with Parkinson's disease. Neurorehabil. Neural Repair 38:9. doi: 10.1177/15459683241268556 39104198

[B24] Langeskov-Christensen M. Franzén E. Grøndahl Hvid L. Dalgas U. (2024). Exercise as medicine in Parkinson's disease. J. Neurol. Neurosurg Psychiatry 95, 1077–1088. doi: 10.1136/jnnp-2023-332974 38418216

[B25] Lavy S. Melamed E. Cooper G. Bentin S. Rinot Y. (1979). Regional cerebral blood flow in patients with Parkinson's disease. Arch. Neurol. 36, 344–348. doi: 10.1001/archneur.1979.00500420054005 454231

[B26] Li T. Wang L. Piao Z. Chen K. Yu X. Wen Q. . (2023). Altered neurovascular coupling for multidisciplinary intensive rehabilitation in Parkinson's disease. J. Neurosci. 43, 1256–1266. doi: 10.1523/JNEUROSCI.1204-22.2023 36609454 PMC9962778

[B27] Liao Y. Y. Chen I. H. Hsu W. C. Tseng H. Y. Wang R. Y. (2021). Effect of exergaming vs. combined exercise on cognitive function and brain activation in frail older adults: a randomised controlled trial. Ann. Phys. Rehabil. Med. 64:101492. doi: 10.1016/j.rehab.2021.101492 33454398

[B28] Luthra N. S. Mehta N. Munoz M. J. Fantuzzi G. Lamotte G. Haus J. M. . (2025). Aerobic exercise-induced changes in fluid biomarkers in Parkinson's disease. NPJ Parkinsons Dis. 11, 190. doi: 10.1038/s41531-025-01042-8 40595707 PMC12215721

[B29] Maidan I. Nieuwhof F. Bernad-Elazari H. Bloem B. R. Giladi N. Hausdorff J. M. . (2018). Evidence for differential effects of 2 forms of exercise on prefrontal plasticity during walking in Parkinson's disease. Neurorehabil Neural Repair 32, 200–208. doi: 10.1177/1545968318763750 29546797

[B30] Maidan I. Nieuwhof F. Bernad-Elazari H. Reelick M. F. Bloem B. R. Giladi N. . (2016). The role of the frontal lobe in complex walking among patients with Parkinson's disease and healthy older adults: an fNIRS study. Neurorehabil. Neural Repair 30, 963–971. doi: 10.1177/1545968316650426 27221042

[B31] Marras C. Beck J. C. Bower J. H. Roberts E. Ritz B. Ross G. W. . (2018). Prevalence of Parkinson's disease across North America. NPJ Parkinson's Disease 4:21. doi: 10.1038/s41531-018-0058-0 30003140 PMC6039505

[B32] McKee K. E. Hackney M. E. (2013). The effects of adapted tango on spatial cognition and disease severity in Parkinson's disease. J. Mot. Behav. 45, 519–529. doi: 10.1080/00222895.2013.834288 24116748 PMC3864026

[B33] Mera T. O. Heldman D. A. Espay A. J. Payne M. Giuffrida J. P. (2012). Feasibility of home-based automated Parkinson's disease motor assessment. J. Neurosci. Methods 203, 152–156. doi: 10.1016/j.jneumeth.2011.09.019 21978487 PMC3221741

[B34] Mitchell A. K. Bliss R. R. Church F. C. (2024). Exercise, Neuroprotective Exerkines, and Parkinson's disease: a narrative review. Biomolecules 14:1241. doi: 10.3390/biom14101241 39456173 PMC11506540

[B35] Mohammadi-Abdar H. Ridgel A. L. Discenzo F. M. Loparo K. A. (2016a). Design and development of a smart exercise bike for motor rehabilitation in individuals with Parkinson's disease. IEEE ASME Trans. Mechatron. 21, 1650–1658. doi: 10.1109/TMECH.2015.2508030 27298575 PMC4902297

[B36] Mohammadi-Abdar H. Ridgel A. L. Discenzo F. M. Phillips R. S. Walter B. L. Loparo K. A. (2016b). Test and validation of a smart exercise bike for motor rehabilitation in individuals with Parkinson's disease. IEEE Trans. Neural. Syst. Rehabil. Eng. 24, 1254–1264. doi: 10.1109/TNSRE.2016.2549030 27046905 PMC5578867

[B37] Monchi O. Petrides M. Doyon J. Postuma R. B. Worsley K. Dagher A. (2004). Neural bases of set-shifting deficits in Parkinson's disease. J. Neurosci. 24, 702–710. doi: 10.1523/JNEUROSCI.4860-03.2004 14736856 PMC6729250

[B38] Moriarty T. Bourbeau K. Bellovary B. Zuhl M. N. (2019). Exercise intensity influences prefrontal cortex oxygenation during cognitive testing. Behav. Sci. 9:83. doi: 10.3390/bs9080083 31357450 PMC6721405

[B39] Moriarty T. A. Bourbeau K. Mermier C. Kravitz L. Gibson A. Beltz N. . (2020). Exercise-based cardiac rehabilitation improves cognitive function among patients with cardiovascular disease. J. Cardiopulm. Rehabil. Prev. 40, 407–413. doi: 10.1097/HCR.0000000000000545 32947322

[B40] Padilha C. Souza R. Grossl F. S. Gauer A. P. M. de Sá C. A. Rodrigues-Junior S. A. (2023). Physical exercise and its effects on people with Parkinson's disease: umbrella review. PLoS One 18:e0293826. doi: 10.1371/journal.pone.0293826 37917715 PMC10621990

[B41] Pelicioni P. H. S. Lord S. R. Okubo Y. Sturnieks D. L. Menant J. C. (2020). People with Parkinson's disease exhibit reduced cognitive and motor cortical activity when undertaking complex stepping tasks requiring inhibitory control. Neurorehabil. Neural. Repair 34, 1088–1098. doi: 10.1177/1545968320969943 33155508

[B42] Pelizzari L. Di Tella S. Rossetto F. Laganà M. M. Bergsland N. Pirastru A. . (2020). Parietal perfusion alterations in Parkinson's disease patients without dementia. Front. Neurol. 11:562. doi: 10.3389/fneur.2020.00562 32655485 PMC7324722

[B43] Picelli A. Varalta V. Melotti C. Zatezalo V. Fonte C. Amato S. . (2016). Effects of treadmill training on cognitive and motor features of patients with mild to moderate Parkinson's disease: a pilot, single-blind, randomized controlled trial. Funct. Neurol. 31, 25–31. doi: 10.11138/FNeur/2016.31.1.025 27027891 PMC4819815

[B44] Pinti P. Tachtsidis I. Hamilton A. Hirsch J. Aichelburg C. Gilbert S. . (2020). The present and future use of functional near-infrared spectroscopy (fNIRS) for cognitive neuroscience. Ann. N. Y. Acad. Sci. 1464, 5–29. doi: 10.1111/nyas.13948 30085354 PMC6367070

[B45] Possin K. L. Filoteo J. V. Song D. D. Salmon D. P. (2008). Spatial and object working memory deficits in Parkinson's disease are due to impairment in different underlying processes. Neuropsychology 22, 585–595. doi: 10.1037/a0012613 18763878 PMC2862562

[B46] Richman J. S. Moorman J. R. (2000). Physiological time-series analysis using approximate entropy and sample entropy. Am. J. Physiol. Heart Circ. Physiol. 278, H2039–2049. doi: 10.1152/ajpheart.2000.278.6.H2039 10843903

[B47] Ridgel A. L. Abdar H. M. Alberts J. L. Discenzo F. M. Loparo K. A. (2013). Variability in cadence during forced cycling predicts motor improvement in individuals with Parkinson's disease. IEEE Trans. Neural. Syst. Rehabil. Eng. 21, 481–489. doi: 10.1109/TNSRE.2012.2225448 23144045 PMC4139093

[B48] Ridgel A. L. Ault D. L. (2019). High-cadence cycling promotes sustained improvement in bradykinesia, rigidity, and mobility in individuals with mild-moderate Parkinson's disease. Parkinson's Disease 2019:4076862. doi: 10.1155/2019/4076862 30944720 PMC6421744

[B49] Ridgel A. L. Peacock C. A. Fickes E. J. Kim C.-H. (2012). Active-assisted cycling improves tremor and bradykinesia in Parkinson's Disease. Arch. Phys. Med. Rehabil. 93, 2049–2054. doi: 10.1016/j.apmr.2012.05.015 22659536

[B50] Ridgel A. L. Phillips R. S. Walter B. L. Discenzo F. M. Loparo K. A. (2015). Dynamic high-cadence cycling improves motor symptoms in Parkinson's disease. Front. Neurol. 6:194. doi: 10.3389/fneur.2015.00194 26388836 PMC4557094

[B51] Ridgel A. L. Vitek J. L. Alberts J. L. (2009). Forced, not voluntary, exercise improves motor function in Parkinson's disease patients. Neurorehabil. Neural Repair 23, 600–608. doi: 10.1177/1545968308328726 19131578

[B52] Schejter-Margalit T. Kizony R. Ben-Binyamin N. Hacham R. Thaler A. Maidan I. . (2022). Neural activation in the prefrontal cortex during the digital clock drawing test measured with functional near-infrared spectroscopy in early stage Parkinson's disease. Parkinsonism. Relat. Disord. 105, 9–14. doi: 10.1016/j.parkreldis.2022.10.021 36327601

[B53] Shin H. Kim R. Park K. Byun K. (2024). Role of exercise in modulating prefrontal cortical activation for improved gait and cognition in Parkinson's disease patients. Phys. Act. Nutr. 28, 37–44. doi: 10.20463/pan.2024.0006 38719465 PMC11079376

[B54] Skorvanek M. Martinez-Martin P. Kovacs N. Rodriguez-Violante M. Corvol J. C. Taba P. . (2017). Differences in MDS-UPDRS scores based on hoehn and yahr stage and disease duration. Mov. Disord. Clin. Pract. 4, 536–544. doi: 10.1002/mdc3.12476 30363418 PMC6174385

[B55] Stuart S. Belluscio V. Quinn J. F. Mancini M. (2019). Pre-frontal cortical activity during walking and turning is reliable and differentiates across young, older adults and people with Parkinson's disease. Front. Neurol. 10:536. doi: 10.3389/fneur.2019.00536 31191434 PMC6540937

[B56] Su D. Cui Y. He C. Yin P. Bai R. Zhu J. . (2025). Projections for prevalence of Parkinson's disease and its driving factors in 195 countries and territories to 2050: modelling study of global burden of disease study 2021. BMJ 388:e080952. doi: 10.1136/bmj-2024-080952 40044233 PMC11881235

[B57] Thumm P. C. Maidan I. Brozgol M. Shustak S. Gazit E. Shema Shiratzki S. . (2018). Treadmill walking reduces pre-frontal activation in patients with Parkinson's disease. Gait Posture 62, 384–387. doi: 10.1016/j.gaitpost.2018.03.041 29626840

[B58] Tombaugh T. N. (2004). Trail making test A and B: normative data stratified by age and education. Arch. Clin. Neuropsychol. 19, 203–214. doi: 10.1016/S0887-6177(03)00039-8 15010086

[B59] Wang Z. Niu C. Duan Y. Yang H. Mi J. Liu C. . (2024). Research on the application of functional near-infrared spectroscopy in differentiating subjective cognitive decline and mild cognitive impairment. Front. Aging Neurosci. 16:1469620. doi: 10.3389/fnagi.2024.1469620 39777048 PMC11703808

[B60] Willis A. W. Roberts E. Beck J. C. Fiske B. Ross W. Savica R. . (2022). Incidence of Parkinson disease in North America. NPJ Parkinson's Dis. 8:170. doi: 10.1038/s41531-022-00410-y 36522332 PMC9755252

[B61] Yang Y. Fu X. Zhang H. Ouyang G. Lin S. C. (2023). The effect of home-based exercise on motor symptoms, quality of life and functional performance in Parkinson's disease: a systematic review and meta-analysis. BMC Geriatr. 23:873. doi: 10.1186/s12877-023-04595-6 38114897 PMC10731835

